# Prostate Artery Embolization for Lower Urinary Tract Symptoms in Men Unfit for Surgery

**DOI:** 10.3390/diagnostics9020046

**Published:** 2019-04-25

**Authors:** Brian Malling, Lars Lönn, Ruben Juhl Jensen, Mats Lindh, Susanne Frevert, Klaus Brasso, Martin Andreas Røder

**Affiliations:** 1 Department of Diagnostic Radiology, Rigshospitalet, Blegdamsvej 9, 2100 Copenhagen, Denmark; lars.birger.loenn@regionh.dk (L.L.); ruben.juhl.jensen.02@regionh.dk (R.J.J.); Mats.Haakan.Lindh@regionh.dk (M.L.); Susanne.Christiansen.Frevert@regionh.dk (S.F.); 2 Department of Urology, Rigshospitalet, Blegdamsvej 9, 2100 Copenhagen, Denmark; klaus.brasso@regionh.dk (K.B.); Martin.Andreas.Roeder@regionh.dk (M.A.R.)

**Keywords:** embolization, therapeutic, prostatic hyperplasia, lower urinary tract symptoms, urinary retention, clinical trial, radiology, interventional

## Abstract

Nearly one in three men develop lower urinary tract symptoms (LUTS) and 10% clinically progress despite medication. Transurethral resection of the prostate (TURP) is the reference standard for symptoms refractory to medical treatment. However, some patients cannot tolerate TURP for medical (e.g., comorbidity) or technical (e.g., large prostate) reasons. This study estimated the safety and effect of prostate artery embolization (PAE) in men unfit for surgery. A prospective, single-centre trial including men with LUTS or urinary retention secondary to benign prostatic hyperplasia (BPH) who were unfit for surgery. The primary objective was to treat urinary retention and LUTS. Outcome measures included International Prostate Symptom Score (IPSS), quality of life (IPSS-QoL), International Index of Erectile Function (IIEF-5), prostate volume (PV), prostate-specific antigen (PSA), peak void flow (Qmax), post-void residual (PVR), and complications. A *p*-value < 0.05 was considered statistically significant. Eleven consecutive patients with a mean age of 75.2 (SD ± 8.2) underwent PAE. Catheter removal was successful in 60%. IPSS-QoL improved 4.5 points (95% CI: −5.6; −3.4), and PV was reduced by 26.2 cm^3^ (95% CI: −50.9; −2.3). None of the remaining outcomes changed. No major complications occurred. PAE was effective and safe for LUTS and urinary retention associated with BPH in men unfit for surgery.

## 1. Introduction

The prevalence of benign prostatic hyperplasia (BPH) is up to 60% of men in their 60s, and this increases with age [1,2]. Nearly one in three men with BPH will develop lower urinary tract symptoms (LUTS) which is associated with impaired health-related quality of life [3,4]. Medical therapy reduces the risk of clinical progression, but one in ten patients will still experience worsening of LUTS, acute urinary retention, recurrent urinary tract infection or renal insufficiency [5]. Transurethral resection of the prostate (TURP) is the reference standard surgical treatment for patients with symptoms refractory to medical therapy [6,7]. TURP significantly improves symptom scores and uroflowmetry measures, but alternative procedures should be considered in medically complicated patients (e.g., those receiving anti-coagulation treatment), and men with prostate glands larger than 80 mL [6,7]. Complications associated with TURP include the need for blood transfusions, surgical revision, urethral stricture, bladder neck contracture, urinary incontinence, erectile dysfunction, and retrograde ejaculation [8,9]. The risk of complications increases with age and comorbidity which complicates the treatment of especially elderly patients who are the most likely to suffer from complications [1,10,11].

Prostate artery embolization (PAE) is a minimally invasive endovascular procedure recently recommended for LUTS caused by BPH by the National Institute of Clinical Excellence [12]. Several randomized controlled trials have demonstrated that the improvement of the International Prostate Symptom Score (IPSS) after PAE is comparable to that seen after TURP [13,14,15]. The primary mechanism of action is occlusion of the blood supply to the prostate inducing ischemia, subsequent necrosis, and shrinkage of the prostate-relieving bladder-outlet obstruction [16]. The treatment is performed under local anaesthesia, which addresses some of the limitations of traditional surgery as it is well-tolerated by elderly and comorbid patients with large glands [17,18,19].

The purpose of this study was to estimate the efficacy and safety of PAE on LUTS and urinary retention in men unfit for surgery.

## 2. Materials and Methods

### 2.1. Study Design

A prospective, single-centre trial was conducted by the Department of Urology and Diagnostic Radiology at the National Hospital of Denmark from July 2017 to July 2018. The protocol was registered online at ClinicalTrials.gov with identifier NCT03099421. The trial was approved by the regional scientific ethical committee (no. H-17000714, 22 March 2017) and conducted according to the World Medical Association Declaration of Helsinki. Data management was approved by the Danish Data Protection Agency (I-suite no. 05309, 20 February 2017) and performed using a research electronic data-capture software (REDCap) [20]. Informed written and verbal consent was obtained from all the male participants in the study.

### 2.2. Study Population

Participants were enrolled from the uptake area of the capital region of Denmark by an experienced urologist in the hospital setting. The inclusion criteria were permanent or intermittent catheterization or moderate to severe LUTS (IPSS > 7) or impaired quality of life (IPSS-QoL ≥ 3) associated with BPH. Only men who were not candidates for surgery due to technical considerations (e.g., large prostates) or comorbidity were enrolled to reflect challenging cases. The exclusion criteria were known bladder dysfunction, urethral strictures, bladder neck contractures, sphincter anomalies, large bladder diverticulum or stones, renal insufficiency (eGFR < 45 mL/min), urological malignancy, coagulation disturbances, severe atherosclerosis or tortuosity measured on computed tomography angiography (CTA), allergies to contrast medium or contraindication to magnetic resonance imaging (MRI) e.g., a pacemaker.

### 2.3. PAE Technique

Three interventional radiologists who had been introduced to the procedure and with more than 50 years’ combined experience performed all procedures. A pre-procedural CTA was used to evaluate the vasculature and plan the procedure.

On the morning of the procedure, a 14 F Foley balloon catheter was placed (or replaced in men with an indwelling catheter) and filled with a solution of 30% contrast medium and 70% saline to act as an anatomical landmark during angiography. Antibiotic prophylaxis consisted of a single intravenous injection of 1.5 g cefuroxime before, and 200 mg trimethoprim orally twice a day for three days after treatment in accordance with local recommendations.

**Figure 1:**
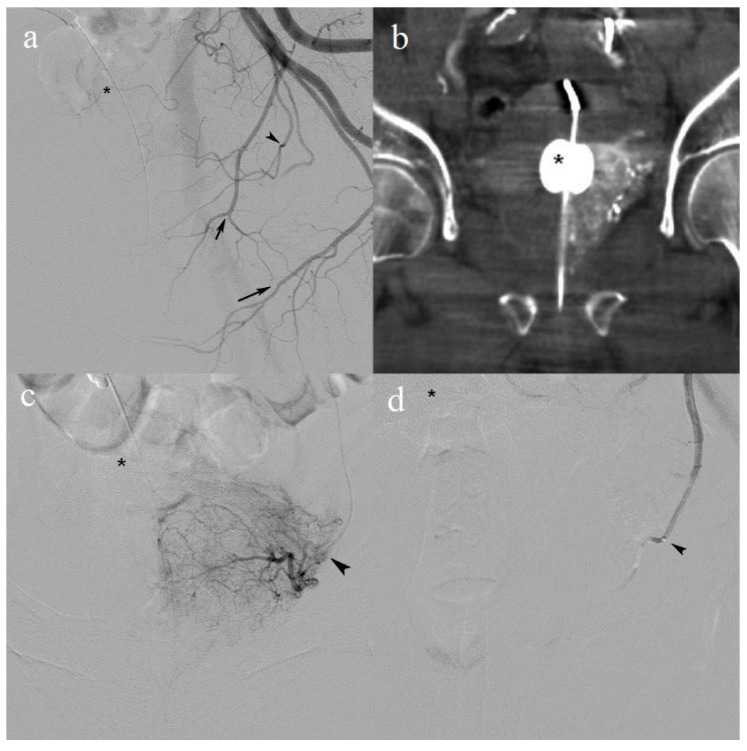
(**a**) Selective digital subtraction angiography (DSA) in the left anterior oblique position with the catheter tip in the left internal iliac artery (not visible). The internal pudendal artery (long arrow) and the characteristic division of obturator artery (short arrow). The prostate artery (arrowhead) protrudes beneath the Foley balloon (*) catheter; (**b**) coronal cone-beam computed tomography with contrast enhancement in the left lobe confirming correct catheter placement; (**c**) frontal DSA before embolization with injection on the microcatheter in the prostate artery (arrowhead); (**d**) frontal DSA with the microcatheter in the same position (arrowhead) and the angiographic endpoint after embolization.

The procedures were performed under local anaesthesia applying a unilateral common femoral artery approach, and a 5F sheath deployment. The prostatic arteries were then localized and super-selectively catheterized with 2.0 F microcatheters applying the PErFecTED—proximal embolization first, then, the embolize distal technique [21]. Intra-arterial nitro-glycerine was used to prevent vessel spasm during microcatheter manoeuvering. Cone-beam computed tomography confirmed catheter placement and ruled out collaterals before manually injecting 300–500 µm tris-acryl microspheres particles under fluoroscopic guidance until stasis, see Figure 1a–d. Every mL of particles administered was followed by three mL of saline.

The embolization technique, duration, fluoroscopy time, radiation exposure, technical outcome (uni- or bilateral embolization), iodine contrast medium, and the volume of particles administered were registered. Technical success was defined as bilateral embolization because of the greater potential for clinical success compared to unilateral embolization [22].

Men experiencing post-embolization syndrome were prescribed 1 g of paracetamol four times a day and 400 mg of a non-steroid anti-inflammatory drug (Ibuprofen) three times a day. An overnight hospital stay allowed for observation and 24 h post-treatment blood sampling. The Foley catheter was removed before discharge in men without previous urinary retention.

### 2.4. Outcome Measures

All participants underwent a medical review including pre-procedural CTA, and a magnetic resonance imaging (MRI) scan at baseline, one and six months after treatment. The primary outcome in men with urinary retention was the ability to void six months after treatment. Mean difference of the international prostate symptom score (IPSS) with a quality of life (IPSS-QoL) scale from 0 (delighted) to 6 (terrible) answering the question “If you were to spend the rest of your life with your urinary condition just the way it is now, how would you feel about that?” was the primary outcome in men with LUTS [23]. Erectile function was assessed using the 5-item international index of erectile function (IIEF-5) questionnaire on a score from 5 (severe erectile dysfunction) to 25 (no erectile dysfunction). Post-void residual (PVR) and peak urinary flow rate (Qmax) were measured in men who were able to void spontaneously. Blood sampling including prostate-specific antigen (PSA) was performed in all men before and 24 h, one and six months after the procedure.

**Figure 2:**
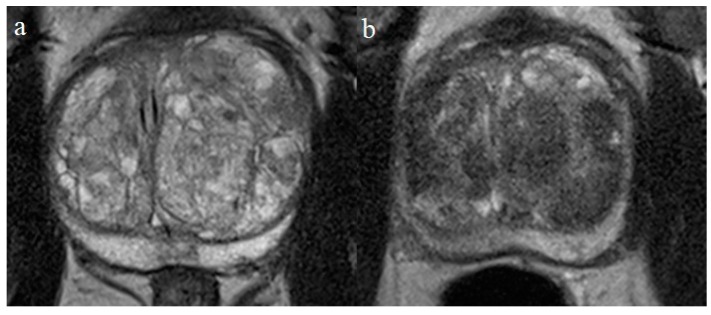
(**a**) Benign prostatic hyperplasia in a patient demonstrated on magnetic resonance imaging with T2-weighted turbo spin echo sequence in the transversal plane. Notice the adenoma in the left lobe compressing the prostatic urethra; (**b**) the same patient three months after treatment with a reduced T2 signal corresponding to infarction of the adenoma.

The MRI protocol used for prostate volume (PV) measures included a T2-weighted turbo spin echo sequence in the transversal and sagittal plane, performed on a 3-Tesla scanner using a phased-array 32-channel body coil, see Figure 2a,b. The U.S Food and Drug Administration (FDA) certified edition of OsiriX imaging software (version 10.0.2, Pixmeo, Geneva, Switzerland) was used to measure PV by drawing the contour of the prostate on all slices [24].

Clinical success was defined as a ≥3-point reduction in IPSS after the cessation of medical therapy. In men with urinary retention, success was the ability to void after catheter removal. Safety was assessed by recording complications classified as minor or major depending on the outcome according to the recommendations of the Society of Interventional Radiology [25]. Pelvic discomfort, urethral pain, and self-limited worsening of voiding symptoms were recorded as a part of the post-embolization syndrome as proposed elsewhere [26].

### 2.5. Statistical Analysis

The statistical analysis was performed using R statistical software version 3.5.2 for Windows [27]. Categorical (i.e., complications) and continuous variables were reported as the total number of events, and means with standard deviations (SD), respectively. The paired *t*-test for repeated measures was used to calculate changes from baseline, and 95% confidence intervals (CI) were reported. A *p*-value < 0.05 was considered statistically significant.

## 3. Results

Eleven consecutive men with a mean age of 75.2 years (SD ± 8.2) were included. Six men suffered from moderate-severe LUTS with a mean IPSS of 18.7 (SD ± 11.2) despite medical therapy consisting of α-blocker, 5α-reductase inhibitor or a combination of both. The remaining five men suffered from urinary retention and were catheter-dependent for an average of 12.2 months (SD ± 10.5). Testosterone replacement treatment for primary hypogonadism was initiated in one patient who was excluded from PV analysis. Hematuria was present in six men, and two men suffered from recurrent urinary tract infections. Three men had previously undergone TURP, two refused TURP because of the risk of complications, four had large prostates (>80 mL), and the remaining had cardiopulmonary comorbidity. Baseline characteristics are shown in Table 1.

Successful technical outcome (i.e., bilateral embolization) was achieved in all procedures, and the PErFecTED technique was used in eight men. Due to atherosclerosis, it was not possible to advance the microcatheter in two cases and unattainable in the remaining case because of penile collateral.

The procedure and fluoroscopy time were 153 min (SD ± 30) and 48 min (SD ± 13), respectively. Patients were exposed to a dose area product of 1054 Gy∙cm^2^ (SD ± 622) and received a 129 mL (SD ± 40) contrast medium. A mean volume of 5 mL (SD ± 3) of particle suspension was injected per procedure.

Clinical success, i.e., the ability to void after catheter removal was 60%, and all six men (100%) without urinary retention reported improved IPSS and IPSS-QoL. Mean IPSS-QoL improved significantly by 4.5 points (95% CI: −5.6; −3.4), but the 11.3-point reduction in IPSS was not significant (*p*-value 0.07). PV was reduced by 26.6 cm^3^ (95% CI: −50.9; −2.3), and PSA was significantly elevated 24 h post-treatment. Hematuria had resolved in four out of six men. None of the remaining outcomes changed significantly, see Table 2.

No major complications occurred. Three cases of minor complications were urinary tract infection (*n* = 1) treated with one week orally administered antibiotics (*n* = 1), and perforation during microcatheter manoeuvring in a distal branch of the prostate artery (*n* = 2). The perforation was embolized using a histoacryl/lipiodol mixture in one case, and by placing a coil in the remaining. Post-embolization syndrome, i.e., urethral pain, pelvic discomfort and frequent urination were reported by four men.

## 4. Discussion

The safety and effect of PAE on LUTS in men with BPH has been established in recent years [28]. However, in this study, only challenging clinical cases who were unfit for urological surgery were enrolled. With a mean age of 75 years, this population represented elderly males, and even though age is not a contradiction for urological surgical intervention, it is reasonable to presume a higher prevalence of comorbidities and large prostate volumes due to the progressive nature of BPH. Considering the expected demographic shift where more people live longer, it is paramount to develop minimally invasive therapies filling the gap between inadequate medical therapy and surgery. Especially since almost one in three men in their 70s with mild LUTS will experience acute urinary retention within 10 years [29].

Our findings suggest that PAE is a viable treatment even in men who would not otherwise tolerate surgery. The 60% chance of a successful trial without catheter confirmed the results from a study investigating the effect on urinary retention on a similar population of poor surgical candidates [17]. Barry et al. showed that a three-point improvement in the symptom score was needed for the patient to perceive being “slightly improved” [30]. Accordingly, IPSS-QoL significantly improved in all patients who suffered from LUTS, reporting improvement above or equal to the three-point minimal clinically important difference. On the other hand, IPSS was not significantly improved (*p*-value of 0.07). Discontinuation of 5α-reductase inhibitor, α-blocker or both, thus avoiding taking a daily pill, potential side effects and reducing patient costs might explain why IPSS-QoL improved independently of a change in IPSS. Introducing a washout period in future studies would avoid any potential influence on results caused by BPH-related drugs. For instance, regrowth of the prostate after discontinuation of 5α-reductase inhibitor is well-known, but whether active treatment at the time of PAE impacts the effect remains controversial [31,32].

After embolization, a significant increase in PSA was observed. This biochemical response is commonly attributed to prostatic inflammation and ischemia, and values above 75–85 ng/mL after treatment are associated with better symptomatic improvement and a higher volume of infarction in the prostate [19,33,34,35].

More than half of the men suffered from gross haematuria which resolved in 67% after embolization, confirming the effect of embolization reported previously [36,37]. Gross haematuria is a debilitating and common condition seen in almost 10% of males presenting with LUTS to the emergency department [38]. Despite the lack of evidence, TURP remains the mainstay treatment recommended by international guidelines [6,39]. Future studies could clarify if PAE should be preferred to TURP in men with LUTS and concomitant haematuria of prostatic origin.

There are several limitations of this study, including the non-controlled study design and small sample size with all outcome measures being available in only a few participants. The relatively short follow-up does not address long-term effects including the need for re-treatment.

In conclusion, PAE was an effective and safe treatment for LUTS and urinary retention associated with BPH in men unfit for surgery.

## Figures and Tables

**Table 1:** Baseline characteristics.

Variable	*n*	Mean	SD
Age, years	11	75.2	8.2
BMI	11	26.0	3.6
IPSS	6	18.7	11.2
IPSS-QoL	6	5.3	0.8
IIEF-5	2	16.5	7.8
PVR, mL	4	60.5	71.7
PSA, µg/L	11	11.2	9.3
PV, cm^3^	11	116.6	64.0
Q_max_, mL	4	12.4	8.1
Catheterization, months	5	12.2	10.5

BMI, body mass index; IPSS, international prostate symptom score; IPSS-QoL, quality of life; IIEF-5, international index of erectile function; PVR, post-void residual; PSA, prostate-specific antigen; PV, prostate volume; Q_max_, peak urinary flow rate; SD, standard deviation.

**Table 2:** Change from baseline.

Variable	*n*	Change	95% CI	*p*-Value
IPSS				
1 month	6	−10.2	[−23.1; 2.8]	0.10
6 months	6	−11.3	[−24.1; 1.5]	0.07
IPSS-QoL				
1 month	6	−3.5	[−5.1; −1.9]	<0.01
6 months	6	−4.5	[−5.6; −3.4]	<0.01
IIEF-5				
1 month	2	3.5	[−91.8; 98.8]	0.72
6 months	2	5.5	[−64.4; 75.4]	0.50
PVR, mL				
1 month	4	16.2	[−84.6; 117.1]	0.64
6 months	2	7	[−5.7; 19.7]	0.09
PSA, µg/L				
24-h	10	119.8	[41.0; 198.7]	<0.01
1 month	11	−2.5	[−7.6; 2.6]	0.30
6 months	11	−4.6	[−9.2; 0.0]	0.051
PV, cm^3^				
1 month	10	−17	[−31.3; −2.7]	0.03
6 months	10	−26.6	[−50.9; −2.3]	0.04
Q_max_, mL/s				
1 month	3	3.8	[−5.7; 13.2]	0.23
6 months	2	0.4	[−5.3;6.2]	0.50

CI, confidence interval; IPSS, international prostate symptom score; IPSS-QoL, quality of life; IIEF-5, international index of erectile function; PVR, post-void residual; PSA, prostate-specific antigen; PV, prostate volume; Q_max_, peak urinary flow rate.
